# Evaluation of Marginal Adaptation of Root-End Filling Materials Using Scanning Electron Microscopy

**Published:** 2013-10-07

**Authors:** Helder Fernandes Oliveira, Ana Helena Gonçalves Alencar, José Antônio Poli Figueiredo, Orlando Aguirre Guedes, Daniel de Almeida Decurcio, Carlos Estrela

**Affiliations:** a Federal University of Goias, Goiânia, Brazil; b Pontifical Catholic University of Rio Grande do Sul, Porto Alegre, Brazil; c University of Cuiabá, Cuiabá, Brazil

**Keywords:** Marginal Adaptation, MTA, Root Canal Filling Materials, Scanning Electron Microscopy

## Abstract

**Introduction:**

The importance of perfect apical seal in endodontics, more specifically in periradicular surgery, is the motivation/reason for development of root-end filling materials with favorable physical, chemical and biological characteristics. The aim of this in vitro study was to evaluate the marginal adaptation of root-end filling materials using scanning electron microscopy.

**Materials and Methods:**

Twenty five human maxillary anterior teeth were prepared using a K-File #50 to 1 mm short of the apical foramen and filled with gutta-percha and Sealapex using the lateral compaction technique. The apical 3 mm of the roots were sectioned perpendicularly to the long axis of the teeth. A 3-mm-deep root-end cavity was prepared using ultrasonic tips powered by an Enac ultrasonic unit. The teeth were randomly assigned to five groups according to the materials tested including IRM, amalgam, ProRoot MTA, Super-EBA and Epiphany/Resilon. Root-end cavities were filled with the materials prepared according to the manufacturers’ instructions. The root apices were carefully prepared for sputter coating and later evaluation using Scanning Electron Microscope (SEM). The images of root-end fillings were divided into four quadrants and distributed into five categories according to the level of marginal adaptation between the root-end material and the root canal walls. The Fisher exact test with Bonferroni correction was used for statistical analysis. The level of significance was set at P = 0.005.

**Results:**

SEM images showed the presence of gaps in the root-end filling materials. No significant difference was observed between the tested materials (P > 0.005).

**Conclusion:**

ProRoot MTA, IRM, amalgam, Super-EBA and Epiphany/Resilon showed similar marginal adaptation as root-end filling materials.

## 1. Introduction

Perfect root canal preparation and adequate endodontic sealing, prevent microorganisms and endotoxins from reaching apical and periapical tissues and determine the success of endodontic treatment. Different factors, generally of microbial (intra- and extra-radicular infection) or non-microbial origin (endogenous and exogenous), may be responsible for the failure of endodontic treatment. As characteristic signs of endodontic treatment failure, apical periodontitis and sometimes the post-treatment symptoms, indicate the need for endodontic retreatment or surgical intervention (1).

Despite all advancements in endodontic materials, failure in root canal treatments is still frequent since fundamental objectives of treatment are not followed. Endodontic microbiota and structured biofilm in inaccessible areas seem to be causes of failure in endodontic treatment (1-6).

Carefully performed clinical procedures using well-defined and scientifically sound procedures have made it possible to perform endodontic retreatment using the conventional technique rather than apical surgery. However, after trying all conventional alternatives and evaluating the risks and benefits of a dental implant, periapical surgery may be an alternative (7, 8). Several complications such as root perforation, instrument fracture, root canal calcifications 

**Table 1.** Experimental groups according to the root-end filling materials

Group	Root-end filling materials
**1 (n=5)**	IRM (LD Caulk Division, Dentsply International, Milford, DE)
**2 (n=5)**	Amalgam (Logic+, SDI, Bayswater, Vic, Australia)
**3 (n=5)**	ProRoot MTA (Dentsply, Tulsa Dental, Tulsa, OK)
**4 (n=5)**	Super EBATM (Harry J. Bosworth Company, Skokie, IL)
**5 (n=5)**	Epiphany/ResilonTM (EpiphanyTM, Pentron Clinical Technologies, Wallingford, CT, USA)

**Table 2.** Comparison of root-end filling materials according to degree of marginal adaptation (α = 0.005)

ROOT-END FILLING MATERIALS		P-value
**IRM**	Amalgam	1.0
**IRM**	ProRoot MTA	0.048
**IRM**	Super EBA	1.0
**IRM**	Epiphany / Resilon^TM^	0.143
**Amalgam**	ProRoot MTA	1.0
**Amalgam**	Super EBA	1.0
**Amalgam**	Epiphany / Resilon^TM^	0.029
**ProRoot MTA**	Super EBA	0.167
**ProRoot MTA**	Epiphany / Resilon^TM^	0.008
**Super EBA**	Epiphany / Resilon^TM^	0.029

and anatomic variations may lead to endodontic treatment failure. In some cases, conventional endodontic treatments are not sufficient and surgical endodontic intervention is required. Endodontic surgery should be concerned as a worthy alternative, because it prevents tooth extraction (7, 8).

The steps of periapical surgery include the surgical removing and debridement of pathological lesion, root-end resection, root-end preparation and root-end sealing (7); the seal provided by root-end filling materials determines the success of this type of treatment.

Several root-end filling materials have been studied, such as gutta-percha, amalgam, zinc oxide and eugenol cement, zinc oxide and eugenol-based cements (IRM, Super-EBA), Cavit, composite resins and MTA (2-4, 7-30).

Importance of apical sealing in periradicular surgery defines the development of root-end filling materials. Using different methods, these materials have been tested for physicochemical properties (13-15, 18, 20), microbiological properties (5, 8, 16, 25, 30), biocompatibility (4, 7, 10-12, 14, 17, 19) and apical sealing (2, 3, 13, 15, 22-24, 26-30). Scanning electron microscopy (SEM) has been used to evaluate the marginal adaptation of materials, although there are the inherent limitations to in vitro studies conducted in laboratories (21, 22).

The importance of root-end filling materials for endodontic sealing and treatment success is inevitable. Treatment outcome is negatively affected by material’s failure in marginal adaptation, and also propagation of cracks and spaces in the interface between the material and the dentin walls. These facts can justify the importance of this study in which we used SEM to evaluate the marginal adaptation of root-end filling materials.

## 2. Material and Methods

Twenty-five human maxillary anterior teeth extracted for different reasons were selected. Radiographic inclusion criteria were absence of calcified root canals, internal or external resorption, crack or fracture, root filling and the presence of a fully formed apex.

The teeth were stored on 0.2% thymol solution (Pharmacia Biopharma Ltda, Uberlandia, MG, Brazil), then immersed in 5% sodium hypochlorite (NaOCl) (Fitofarma, Goiânia, GO, Brazil) for 30 min to remove soft tissues covering the roots. The crowns were cut to prepare a standardized 16-mm tooth length from the root apex. After initial radiographs the cervical third of each root canal was enlarged using Gates-Glidden drills (Dentsply Maillefer, Ballaigues, Switzerland) sizes 1 to 3 (ISO tip size #50 up to #90) Canals were prepared using K-Files (Dentsply Maillefer, Ballaigues, Switzerland) up to size #50, 1 mm short of the apical foramen. During instrumentation, the root canals were irrigated with 3 mL of 1% NaOCl at each change of files. Root canals were dried and filled with 17% EDTA (pH 7.2) (Biodinâmica, Ibiporã, PR, Brazil) for 3 min to remove the smear layer. After that, the root canals were irrigated again with 3 mL of 1% NaOCl and dried with paper points (Dentsply Maillefer, Ballaigues, Switzerland).

The root canals were obturated with standard 2% gutta-percha points (Tanari, Manacapuru, AM, Brazil) and the matching sealer (Sealapex, Sybron-Endo, Glendora, CA, USA) using the conventional lateral compaction technique. After that, the teeth were wrapped in wet gauze and placed in an incubator at 37ºC for 24 h for setting the filling materials completely.

The apical 3 mm of roots was sectioned perpendicular to the long axis of the tooth with a high-speed Zecrya drill (Dentsply Maillefer, Ballaigues, Switzerland) under continuous air/water spray. Then, a 3-mm-deep root-end cavity was prepared using DF. 908 ultrasonic tips (Osada Eletric, CO., Osada, Japan) powered by an Enac (Osada Eletric CO., Osada, Japan) ultrasonic unit under continuous irrigation with saline solution. The teeth were randomly assigned to 5 groups of specimen each, according to the materials tested (Table 1). The materials were prepared according to the manufacturers’ instruction, and then the root-end cavities were filled. The teeth were wrapped in wet gauze and placed in an incubator at 37°C for 24 h for the root-end filling materials to set completely. 

**Figure 1.**
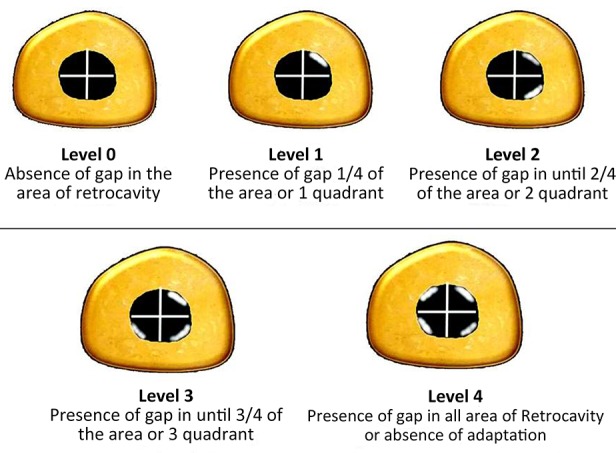
Schematic representation of marginal adaptation of rootend filling materials according to distribution quadrants

The apical 3-mm specimens sectioned from each root were placed in individual plastic vials containing 2.5% sodium hypochlorite solution for 3 h. The specimens were dehydrated for 5 h in increasing concentrations of alcohol (70%, 90% and 99%), then placed on metal stubs, labeled and sputter coated with 150-Å thick gold palladium (MED 020; BAL-TEC, Balzers, Liechtenstein). SEM was performed in the Electronic Microscopy Laboratory of Pontifical Catholic University of Rio Grande do Sul (PUCRS), Porto Alegre, Brazil, using a Philips XL 20. 

Scanning Electron Microscope (Philips, Eindhoven, Netherlands) was operating at 15 KV. Two previously calibrated observers analyzed the images independently.

The root-end area of each specimen was classified according to the presence and extent of marginal gaps into five different degrees: Degree 0 (no marginal gap), degrees 1, 2, 3 and 4 (marginal gap < 1/4 mm, 1/4 -1/2 mm, 1/2-3/4 mm and in the entire area, respectively) (Figure 1). Different types of root-end filling materials were analyzed according to the level of marginal adaptation using the Fisher exact test and SPSS software version 19.0 (SPSS Inc., Chicago, IL, USA). The level of significance was set at α = 0.05. 

## 3. Results

Based on SEM images, the specimens with ProRoot MTA had no significant difference in marginal adaptation compared to those with IRM, amalgam, Super-EBA and Epiphany/Resilon groups (P > 0.005) (Table 2). 

## 4. Discussion

Diagnosis of endodontic failure, directs our attention to alternative treatment options, such as retreatment, endodontic surgery and even tooth extraction. Preventing bacterial leakage was the interest of numerous studies that analyzed the sealing ability of different restorative and root-end filling materials (2, 3, 7, 8, 16, 22).

**Figure 2.**
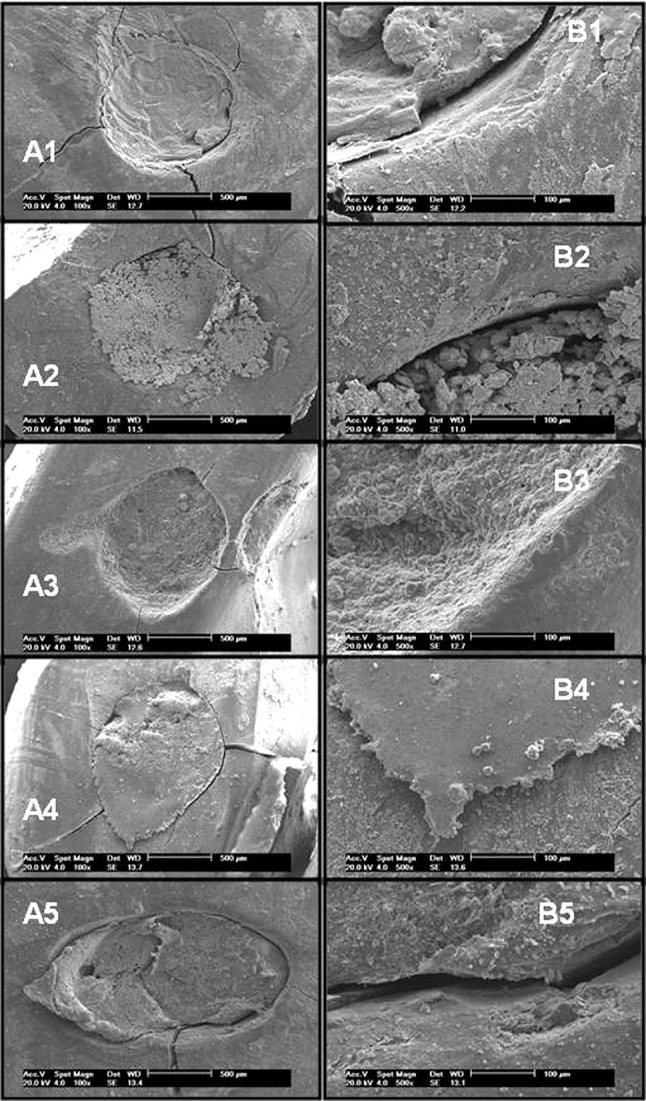
Marginal adaptation in IRM(A1 and B1), amalgam (A2 and B2), ProRootMTA (A3 and B3), Super-EBA (A4 and B4) and Epiphany/Resilon (A5 and B5) at 100× (A) and 500× (B) magnifications

The success of conventional treatment or surgery depends on coronal and apical sealing.

This study evaluated the marginal adaptation of root-end filling materials (IRM, amalgam, ProRoot MTA, Super EBA and Epiphany/Resilon) using SEM images at 100× and 500× magnification. ProRoot MTA produced similar marginal adaptation compared to those found in the IRM, Amalgam, Super-EBA and Epiphany/Resilon groups.

Different methods have been used to evaluate root-end sealing (2, 3, 5, 8, 25, 26). Advantages, viability of studies and limitations inherent to in vitro and in vivo experiments have been discussed. MTA has been evaluated as a sealing material using different methods including infiltration tests (2, 3, 5, 8, 26, 28), fluid transport (27), infiltration with microbial indicators (5, 8, 24, 25, 29, 30), biocompatibility tests (4, 10-12, 19) , and analysis of marginal adaptation using SEM (13, 21, 22).

Torabinejad et al. compared the sealing ability of MTA with silver amalgam and IRM in human teeth by analyzing the penetration of Rhodamine B fluorescent (3). MTA provided excellent marginal sealing and was superior to Super-EBA, which resulted in less infiltration than silver amalgam. Bernabe et al. compared the effect of MTA, IRM, Super-EBA, glass ionomer and silver amalgam with varnish used as root-end filling materials (7). MTA was the material that had the lowest rates of infiltration, whereas IRM had the worst results. The other root-end filling materials had similar results.

Gonçalves and Bramante verified the absence of significant differences on apical sealing ability of Super-EBA and MTA in four root canal filling techniques (28). Wu et al. used five commonly used or potential root-end filling materials (27). The leakage rates of amalgam and Super-EBA decreased with time, whereas the improved seal of MTA was maintained until the end of the experiment. At 3, 6 and 12 months, both glass ionomer cements and MTA resulted in less leakage than amalgam and Super-EBA groups.

Infiltration tests with biological indicators have been used in numerous studies (8, 16, 25). Torabinejad et al. compared the antimicrobial effect of silver amalgam, zinc oxide and eugenol, Super-EBA and MTA and found that no material under their study had antimicrobial activity against strict anaerobic microorganisms (16). However MTA had some effect on 5 of the 9 types of facultative bacteria. Bernabe et al. assessed the histological response of grey MTA and zinc oxide eugenol (ZOE) as root-end filling materials (7). Grey MTA showed less periapical inflammation and tissue response, even when no root filling or coronal restoration was present.

Magnification has also been used to analyze the surface of materials and tooth structures. Torabinejad et al. compared the marginal adaptation of MTA with commonly used root-end filling materials using SEM (13). MTA resulted in better adaptation than amalgam, Super-EBA and IRM. Peters and Peters evaluated the marginal adaptation provided by Super-EBA (EBA) and ProRoot MTA (MTA) in root-end fillings and the occurrence of micro cracks before and after occlusal loadings (21). Both EBA and MTA had excellent marginal adaptation before masticatory loading. After loading, the amount of continuous margin for both root-end filling materials decreased slightly, but was still high. Xavier et al. evaluated the sealing ability of MTA-Angelus, Super-EBA and Vitremer (22). There were significant differences between the three materials. The greatest microleakage was found in the Vitremer group. SEM analysis revealed variable gaps between materials and the dentin walls, and fewer gaps were found in the MTA group.

The result of the present study regarding marginal adaptation of MTA, was similar to some other studies (13, 21, 22). In some SEM images the Epiphany/Resilon group revealed gaps, which is in agreement with the findings reported by Tay et al. (23), and Hollanda et al. (24). Shipper et al. (29) and Maltezos et al. (29) compared the apical sealing ability of Resilon/Epiphany with ProRoot MTA and Super EBA. They found no statistically significant difference between the results produced by Resilon/Epiphany and ProRoot MTA.

SEM analysis showed sealing ability between the materials tested. The biological results (4, 10, 19) and the good marginal adaptation observed by SEM images revealed the potential of ProRoot MTA as root-end filling. However, other studies should be conducted to rank the methods available for these analyses.

## 5. Conclusion

Based on this in vitro study, ProRoot MTA, IRM, amalgam, Super-EBA and Epiphany/Resilon showed similar marginal adaptation when used as root-end filling.
